# Heterotic Trait Locus (HTL) Mapping Identifies Intra-Locus Interactions That Underlie Reproductive Hybrid Vigor in *Sorghum bicolor*

**DOI:** 10.1371/journal.pone.0038993

**Published:** 2012-06-25

**Authors:** Imri Ben-Israel, Benjamin Kilian, Habte Nida, Eyal Fridman

**Affiliations:** 1 The Robert H. Smith Institute of Plant Sciences and Genetics in Agriculture, Faculty of Agriculture, Food and Environment, The Hebrew University of Jerusalem, Israel; 2 Institute of Plant Genetics and Crop Plant Research (IPK), Gatersleben, Germany; New Mexico State University, United States of America

## Abstract

Identifying intra-locus interactions underlying heterotic variation among whole-genome hybrids is a key to understanding mechanisms of heterosis and exploiting it for crop and livestock improvement. In this study, we present the development and first use of the heterotic trait locus (HTL) mapping approach to associate specific intra-locus interactions with an overdominant heterotic mode of inheritance in a diallel population using *Sorghum bicolor* as the model. This method combines the advantages of ample genetic diversity and the possibility of studying non-additive inheritance. Furthermore, this design enables dissecting the latter to identify specific intra-locus interactions. We identified three HTLs (3.5% of loci tested) with synergistic intra-locus effects on overdominant grain yield heterosis in 2 years of field trials. These loci account for 19.0% of the heterotic variation, including a significant interaction found between two of them. Moreover, analysis of one of these loci (*hDPW4.1*) in a consecutive F2 population confirmed a significant 21% increase in grain yield of heterozygous vs. homozygous plants in this locus. Notably, two of the three HTLs for grain yield are in synteny with previously reported overdominant quantitative trait loci for grain yield in maize. A mechanism for the reproductive heterosis found in this study is suggested, in which grain yield increase is achieved by releasing the compensatory tradeoffs between biomass and reproductive output, and between seed number and weight. These results highlight the power of analyzing a diverse set of inbreds and their hybrids for unraveling hitherto unknown allelic interactions mediating heterosis.

## Introduction

Associating of causal polymorphism with complex phenotypes can provide a better understanding of the mechanisms underlying developmental and biochemical constraints, thus enabling accelerated crop improvement. Advances in genotyping technologies and computational approaches have reshaped the way genetic analysis is conducted and have allowed capturing a substantial proportion of the global genetic diversity for genome-wide association studies [1]. While different “association mapping” approaches in plants are focused mainly on additive variation between inbred lines, much less attention has been paid to developing experimental and computational approaches for identifying and deciphering the possible role of intra-locus interactions that contribute to the phenotypic landscape of the hybrids. The underlying genetics of heterosis has long been debated–ever since it was first observed and documented by Charles Darwin [2] and later studied experimentally with natural and artificial populations of various organisms. Although it is agreed that increased homozygosity often lowers fitness-related characters (survival, growth rate and fertility), at the heart of the debate is the extent to which this can be attributed to increased homozygosity for partially recessive detrimental mutations (dominance), rather than changes in homozygosity for alleles at loci with heterozygote advantage (overdominance model [3]). This debate also holds for agricultural yield: is the vigor of the hybrids the outcome of many dominant loci with intermediate effects working in a multiplicative manner on different yield-associated traits, or are those overdominant loci key regulators in several pathways throughout plant development?

Two recent reports illuminate this debate, although the final conclusion of both may still be linked to the type of plant system and phenotype analyzed, as well as to the genetic background (whole genome hybrids compared to isogenic homozygous background). Riedelsheimer *et al*. [4] crossed 285 diverse Dent inbred lines from worldwide sources with two testers and predicted their combining abilities for seven biomass- and bioenergy-related traits using 56,110 sngle-nucleotide polymorphisms and 130 metabolites. Then they performed genome-wide association scans using a Q+K model [5] and found no strong association signals, even though population size, heritabilities of the traits, extent of linkage disequilibrium and marker density were sufficiently high to detect large quantitative trait loci (QTLs). Based on these results, it was concluded that the genetic architecture that underlies heterosis is close to an infinitesimal model. On the other hand, Krieger *et al*. [6] searched for genes that cause heterosis in tomato (*Solanum lycopersicum)* by crossing 33 diverse fertile mutants with the matching non-mutagenized parent known as ‘M82’ to create isogenic mutant heterozygotes. Comparison of their yield traits identified six mutant heterozygotes that showed yield heterosis, with the individual effects ranging from 36% to 88%.

To date, no individual overdominant locus has been isolated by unbiased QTL mapping and recent attempts to zoom in on genomic loci associated with heterosis have mostly made use of biparental populations. This is performed by estimating the heterosis phenotype through test-crossing introgression lines or recombinant inbred lines to the recurrent parent [7], [8] and linking the heterotic mode of inheritance with marker-defined genetic intervals. Additional approaches have included the use of immortalized F2 populations [9] and the fine-mapping of heterotic QTLs in nearly isogenic lines [10]. Lariepe *et al*. [11] recently reviewed the use of biparental populations for dissecting heterosis and extending the use of the North Carolina III (NCIII) design with markers [12] to develop and implement a multiparental-connected model. This allowed studying heterosis in families derived from both related and unrelated parents, and comparing not only contrasts between homozygous and heterozygous genotypes, but also contrasts between heterozygous genotypes at each locus [11]. One approach for studying heterosis, devised very early in plant and animal breeding, was generation of a cross matrix (diallel) between founder lines (FLs) followed by phenotypic analysis of these inbred lines and their hybrids. Kearsey [13] was the first to compare the merits of five designs–full diallel, half diallel, partial diallel, NCI and NCII. These studies, which were conducted before the molecular concepts of genetics had been formulated, were concerned with dissecting the heterotic variation into its components (additive, dominant and epistatic), as well as with determining general and specific combining abilities between the different founder inbreds. With the advent of genetic marker technologies, it became feasible to estimate the relationship between overall genetic distance and the magnitude of heterosis calculated by the general combining ability of the parents [14]. Cho *et al*. [15] connected genetic heterogeneity and heterosis at each locus by calculating the differences in the levels of best parent heterosis (BPH) between different genotypes. The ANOVA comparison between BPH values of homozygotes and heterozygotes at each locus concluded that more than 30% of the markers were associated with a heterotic mode of inheritance. Note that this analysis did not distinguish between different allelic combinations among the hybrids and treated the heterozygotes in each locus as a single group.

Previous studies have reported a high level of heterosis in *Sorghum bicolor*, the fifth most important crop in the world (www.FAO.org), which exhibits a consistent yield increase in hybrids vs. varieties (up to more than double) under a wide range of growing and management conditions [16], [17]. Interestingly, these early studies led to the identification of one of the few cases of whole-plant heterosis resulting from the heterozygous condition of a single mutation that affects duration of growth [18]. However, since the underlying gene was not identified, the possibility that this non-additive mode of inheritance is due to pseudo-overdominance [7] of more than one gene still remains open. Heterotic groups are not as clearly defined in sorghum as in maize, and studies using molecular markers have shown that prediction of heterosis is enhanced by using particular linkage groups in models attempting to associate genetic distance and hybrid-group performance [19]. Although these observations lend some support to the attractive possibility of identifying single genes underlying heterosis in this major crop plant, the heterosis map of sorghum, which would allow alignment with existing maps in maize [9], [11], [12], [20], rice [21] and other plants, is still lacking.

Here we present the first use of heterotic trait locus (HTL) mapping to identify intra-locus interactions mediating an over- or underdominant mode of inheritance. The wide diallel population of *S. bicolor* is derived from 19 founder lines (FLs) selected from a wide gene pool. This population was phenotyped for 2 consecutive years and statistical and computational tools were integrated to test for association between specific intra-locus interactions and overdominant mode of inheritance. We discuss possible mechanisms underlying heterosis for grain yield in hybrids based on our experimental data and demonstrate that the results of this fast-track mapping are validated by the overdominant mode of inheritance of one HTL in the consecutive F2 population. Finally, we propose the incorporation of individual resequenced genomes of only diallel FLs to zoom in on hitherto unknown loci underlying heterosis, thereby paving the way for an improved understanding of molecular mechanisms underlying this elusive and important phenomenon.

## Materials and Methods

### Sampling, Genotyping and Genetic Analysis of Wide Collection of *S. bicolor*

This study was initiated by obtaining 173 available *S. bicolor* ssp. *bicolor* accessions from the Israeli Plant Gene Bank (IGB; http://igb.agri.gov.il/main/index.pl; Table S1). In addition, the USDA cultivated sorghum collection in the Germplasm Resources Information Network database (GRIN; http://www.ars-grin.gov) was mined and an additional 100 accessions were added. All plants were grown in pots in a greenhouse for DNA extraction and seed collection. DNA was extracted as follows: young leaf samples were ground in tubes using a TissueLyser (Qiagen, Hilden, Germany); 0.7 mL nuclei lysis solution (0.2 M Tris pH 7.5, 0.05 M EDTA, 0.2% w/v CTAB and 2 M NaCl) was added to each sample before incubating at 65°C for 1 h, and then 800 µL chloroform:isoamyl alcohol (24∶1, v/v) was added. Samples were shaken for 15 min and centrifuged for 15 min at 10,000 *g*; the supernatant was transferred to a new tube, and 2.5 µL RNase (Qiagen) was added before incubating at 37°C for 15 min. DNA was precipitated by centrifugation with 500 µL ice-cold isopropanol and the pellet was washed with 0.2 M sodium acetate (pH 5.2) in 76% ethanol at room temperature for 5 min before centrifugation for 5 min at 10,000 *g*. This was followed by an additional wash of the pellet with 10 mM ammonium acetate in 76% ethanol at room temperature for 5 min and centrifugation for 5 min at 10,000 *g*. DNA was dried and dissolved in 30 µL DDW.

Microsatellite [simple sequence repeat (SSR)] genotyping was conducted using multiplexes of three markers with primers labeled with FAM, HEX or TAMARA (Table S2). PCR products were separated and analyzed using the MegaBACE Genetic Profiler and Fragment Profiler software tools (Amersham Biosciences, München, Germany). The raw data were entered into the database and examined for typing errors, false-positive alleles and data authenticity.

Analysis of genetic diversity and allele frequency, phylogenetic tree construction and genetic distances were calculated with PowerMarker version 3.25 [22] as described previously [23]. FLs for the diallel were selected using the “line selection” function in the design tool, in the analysis option, using “allele number” as the major criterion for selection.

### Construction of Diallel and Plant Phenotype

A crossing scheme was designed to allow continuous and synchronous flowering of the FLs. To perform crosses between all FLs, the panicles of the female plant were emasculated by hot-water treatment (45°C for 10 min) with dehiscence control using a plastic bag (Stephens and Quiniby, 1933 in [24]). Non-emasculated flowering panicles were used for manual pollination 1 to 3 days after the hot-water treatment. Prior to the field trials, the hybrid seeds were sown in trays and DNA was extracted from leaves of 2- to 3-week-old seedlings in 96-well plates using the high-throughput method described by Xin *et al*. [25]. This DNA was subjected to high resolution melting (HRM) genotyping on a LightCycler (Roche, Basel, Switzerland) using SSR markers Xtxp57 or Xtxp321 (Table S2). Inbred and artificial hybrid DNA (1∶1 mix of inbreds) was used as a control in each 96-well plate, and HRM conditions were as described previously [26] for validation of hybrids. Hybrids and FLs were transplanted into the experimental farm at the Robert H. Smith Faculty of Agriculture, Food and Environment (Rehovot, Israel) as single plants (15 cm between plants, 80 cm between rows, which is comparable for sorghum density at commercial field [27]) in a complete-randomized block design. In 2010, two FLs and their derived hybrids were excluded from the analysis due severe chemical damage of these FLs during germination in trays. A total of 123 hybrids (out of 136 possible) and 27 reciprocal crosses were analyzed. In 2011, a total of 157 hybrids (out of 171 possible) and 15 reciprocal crosses were analyzed (Figure S1A and S1B). Trials included 7 or 4 replicates per hybrid and 14 or 10 replicates per parent line, respectively.

Number of days to flowering was scored for each plant when half of the panicle was at anthesis. Traits evaluated at harvest were: plant height (from the soil to the base of the panicle); stem diameter (of the lower third of the stem); leaf weight, and stem weight. Panicles were oven-dried for 2 days at 65°C and dry panicle weight (DPW) was measured. Next, the panicle was dissected following Brown *et al*. [28] to determine primary, secondary and tertiary branching number, as well as rachis length, whorl number and primary branch length. Seed dry weight (SDW) was obtained by weighing a total of 50 grains, then seed number (SN) was calculated as SN = (DPW/SDW) x 50.

### Calculation of Overdominant Heterosis (ODH) Value

The ODH parameter of the r progeny of a cross FLx X FLy was determined as follows (P2_xy_ and P1_xy_ are the high and low mean values of the two parents, respectively):

1. If the phenotypic value of replicate r of the cross, F1_xyr_, is greater than or equal to P2_xy_, then the ODH parameter of replicate r, ODH_xyr_, is calculated using the algebraic expression: ODH_xyr_ = (F1_xyr_ - P2_xy_)/P2_xy._
2. If the phenotypic value of the replicate r, F1_xyr,_ is less than or equal to P1_xy_, then ODH_xyr_ is calculated using the algebraic expression: ODH_xyr = _(F1_xyr_ - P1_xy_)/P1_xy._
3. If the phenotypic value of the replicate r, F1_xyr_, is between P1_xy_ and P2_xy_, then ODH_xyr_ = 0.

The mean ODH_xy_ for the cross was then calculated for all replicates of the cross using the algebraic expression 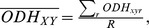 where R is the total number of replicates of the cross.

### Correlation Patterns of Traits and of Heterotic Values

JMP software (SAS Institute, Cary, NC) was used to perform multiple correlation analyses using the “stepwise” option under the “fit model” function. A forward direction was taken to select factors for the final model that correlate between traits or heterosis levels (this test was conducted using the hybrid means).

### HTL Mapping

Data derived from each of the 2 years were analyzed separately. In the first stage, a general linear regression was implemented using TASSEL software [29] to assess the effect of the genetic heterogeneity of each marker on the ODH in the diallel (Figure S2A and S2B). The model was: *Y_ij_ = µ+α_i_+e_ij_*, where *µ* is the mean ODH of the diallel population, *α_i_* is the effect of the genotypic group and *e_ij_* is the random error, i.e. the variation between the ODH means of the different crosses of same genotypic group. The non-distribution-dependent, experiment-wise error level of α = 0.05 was computed based on 1000 permutations [29], [30].

The significant loci were then analyzed in the second step: A non-linear Kolmogorov-Smirnov pair-wise test (R environment http://www.r-project.org/) was used to compare the ODH distribution of the different hetero-genotypic groups to the homozygous group in these loci (Figure S2C and S2D): *D_n_*_,*n*’_ = sup*_x_* [*F*_1,*n*_(*x*) − *F*_2,*n*’_(*x*)], where sup*_x_* is the supermum of the set of distances, and *F*_1,*n*_ and *F*_2,*n*’_ are the empirical ODH distribution function of a specific hetero-genotypic group and the homozygous allelic combinations, respectively. The null hypothesis is rejected at level α if 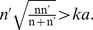


### HTL Mapping with Consideration of the FLs Genomic Structure

Estimation of cluster likelihood was calculated based on five independent runs (STRUCTURE software [31]) for a variable number of clusters, from K = 2 to K = 10 (length of burning period: 20,000 and number of MCMC repeats: 20000). K = 4 was chosen due to the low variation of probability values and repetitive clustering.

For each hybrid, the combination of FLs clustering was determined and this new variable was considered a co-factor in the analysis. The GLM model was *Y_ij_ = µ+α_i_+β_k_+e_ij_*, where *µ* is the mean ODH of the diallel population, *α_i_* is the effect of the genotypic group, *β_k_* is the effect of the clustering assignment of the FLs and *e_ij_* is the random error. For the Kolmogorov-Smirnov test, the ODH values of the hybrids were adjusted, considering the effect of the clustering assignment of the FLs. The effect of each combination was measured as the deviation of this combination from the weighted average of ODH values.

### Three-way and Epistatic Relation Analyses

In each HTL, allelic combinations were assembled into two genetic states: P (positive intra-locus interaction) or N (neutral, other allelic combinations). JMP software (SAS Institute, Cary, NC) was used to perform two- and three-way ANOVA using the “Standard Least Squares” option under the “fit model” function with the EMS method. The two-way model was *Y_ijk = _µ+α_i_+γ_j_+αγ_ij_+β_k_+e_ijk_*, where *µ* is the mean ODH value of the diallel population, *α_i_* and *γ_j_* are the main fixed effects of the HTLs, *αγ_ij_* is the genetic interaction effect, *β_k_* is the random effect of the year, and *e_ijk_* is the random error.

The three-way model was: *Y_ijrk = _µ+α_i_+γ_j_+θ_r+_αγ_ij_+β_k_+e_ijrk_*, where *µ* is the mean ODH value of the diallel population, *α_i_*, *γ_j_* and *θ_r_* are the main fixed effects of the HTLs (*hDPW1.1*, *hDPW1.2* and *hDPW4.1*, respectively), *αγ_ij_* is the effect of interaction between HTLs *hDPW1.1* and *hDPW1.2*; *β_k_* is the random effect of the year, and *e_ijrk_* is the random error.

### Analysis of F2 Population

A single validated hybrid between SB018 and SB153 that carries the 154 and 162 alleles of the Dsenhabm39 SSR marker was selfed to obtain the F2 population. One hundred plants were transplanted into the field during the 2011 trial and phenotyped with the diallel’s plants of this year. Linkage between the Dsenhabm39 locus and the different traits was tested by ANOVA under the “fit Y by X” function, and the “Compare with the best” option under the Compare means.

### Analysis of Sorghum-maize Synteny

The ‘block view’ tool of Symap (http://www.symapdb.org/) was used to align the sorghum HTLs to the syntenic regions in the maize genome which were then assigned to the corresponding genomic bins (http://www.maizegdb.org/). Maize overdominant QTLs found on these bins were assembled to the synteny map by the physical location of the associated markers (http://www.maizegdb.org/).

## Results

### Genetic Analysis of the Wide Collection of *Sorghum bicolor* ssp. *bicolor*

**Figure 1:**
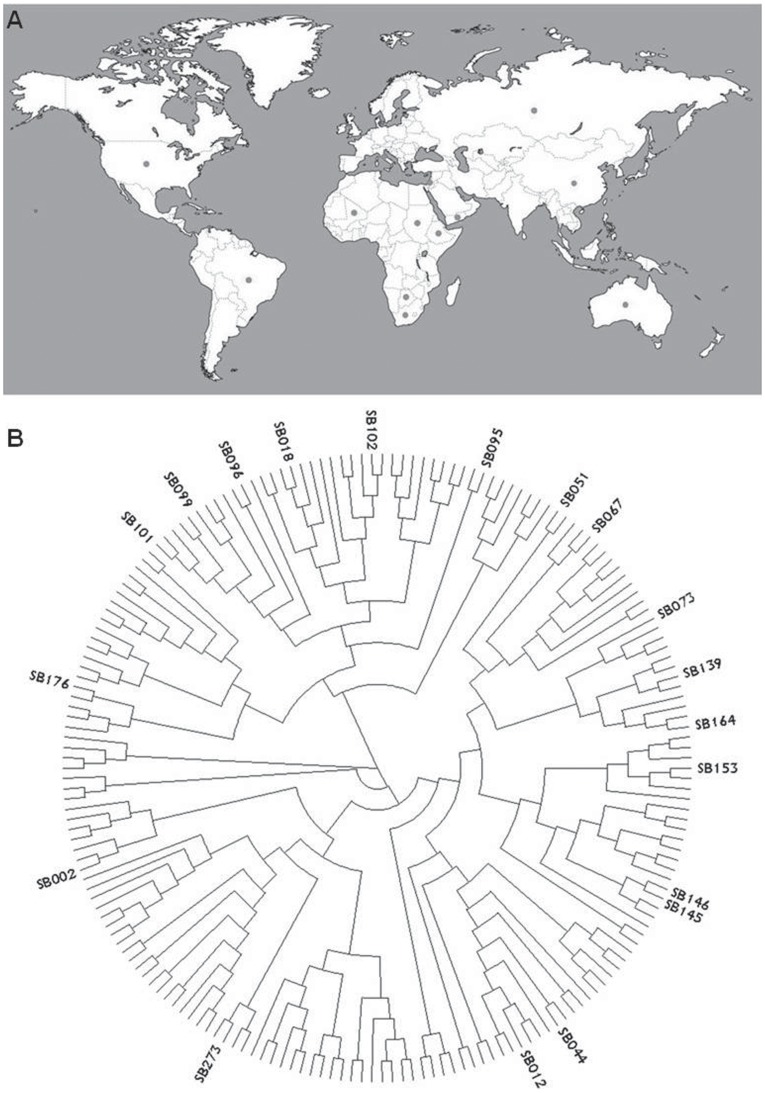
The *Sorghum bicolor* lines used for heterosis mapping. **A.** Origin of the wide collection of lines includes accessions collected worldwide (see Table S1 for details). **B.** Phylogenetic tree of the wide *Sorghum bicolor* ssp. bicolor collection. The external nodes and coding of founder lines (FLs) are indicated.

This study was initiated by obtaining 273 *S. bicolor* accessions, including BTx623 which was used previously for whole-genome sequencing [32]. These accessions originated from different geographical regions worldwide but there were no details of their breeding history (Figure 1A and Table S1). To assess the diversity in this collection, the plants were first genotyped with a set of 50 microsatellite (SSR) markers spanning telomeric and centromeric regions (2 and 1 for each region, respectively [33], [34]; Table S2). This set included 23 EST-derived markers (eSSR) and an additional 27 markers from non-coding genomic regions (gSSR). A subset of 19 FLs was chosen with the goal of harvesting maximum genetic diversity by a reasonable number of homozygous lines, while balancing for equal frequency of alleles in each locus. Such equal frequency allows statistical comparison within the derived diallel mapping population of both heterozygous and homozygous groups in each locus (see discussion). Figure 1B depicts a phylogenetic tree of the original wide collection, including indications for the external nodes of the selected FLs. These FLs represented 58% or 44% of the original allelic diversity based on eSSRs and gSSRs, respectively. On average, based on these marker types, there are 5.7 or 7.5 alleles per locus, respectively. Overall, in each of these loci, there are at least two alleles shared by a minimum of three FLs.

**Figure 2:**
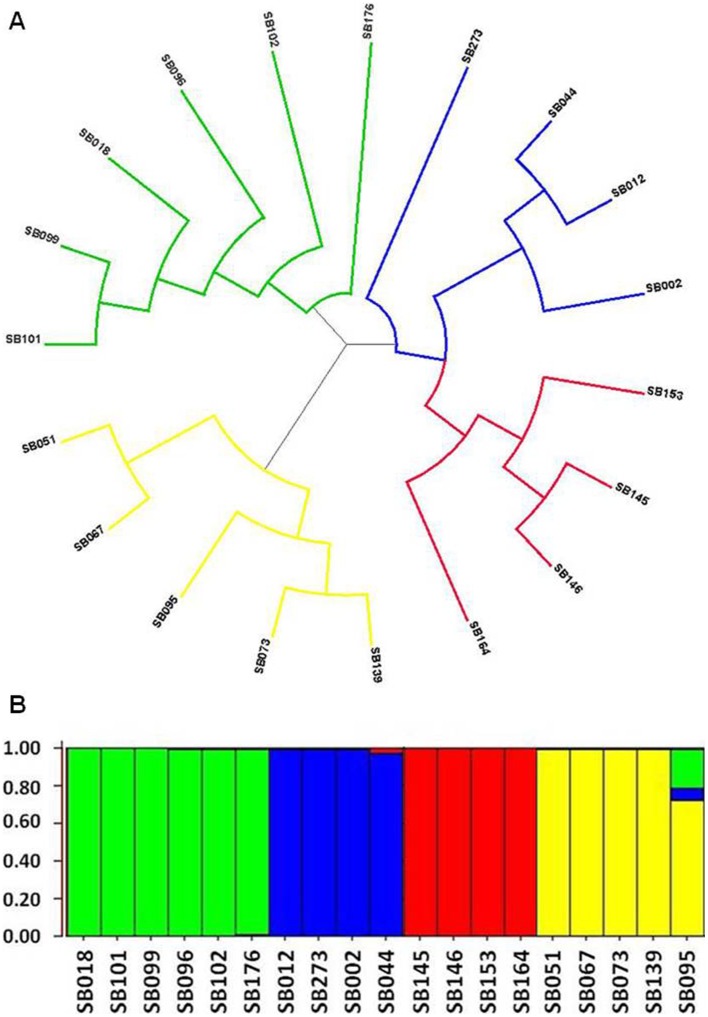
Genetic analysis of the diallel founder lines. **A.** Clustering analysis of the 19 *Sorghum bicolor* inbreds based on unrooted neighbor joining tree. Color coding representing the four identified clusters. **B.** Model-based ancestry for each founder line with enforcement of the cluster number (K) to 4 (see Materials and Methods). Distruct plot is shown with color coding representing the four clusters of the STRUCTURE analysis and the name of each founder line is depicted below.

Next, the genetic analysis of the 19 FLs was supplemented with an additional 35 SSR markers and both Bayesian and genetic distance approaches were taken to further infer the genetic relationship among these inbred lines (see Materials and Methods). Both approaches indicated identical clustering of the FLs into four major genomic groups which were named A, B, C and D (green, blue, red and yellow, respectively; Figure 2A and 2B). Both the fixation index (Fst) values and branching architecture of the phylogenetic tree (Figure 2A) indicated different levels of similarity within each group. Excluding group C, in which lines were relatively clustered (Fst = 0.435), the lines in the other groups were relatively dispersed (Fst = 0.27, 0.27 and 0.21 for groups A, B and D, respectively).

### Analysis of Phenotypic and Heterotic Variation

The FLs selected for the heterosis analysis were intercrossed to achieve a diallel sufficient for two replicated field experiments in 2010 and 2011. The experiments were set up to achieve a reasonable compromise between gaining phenotypic and heterosis values from a large number of hybrids and growing plants under conditions resembling common practice for sorghum under dense planting conditions ([27]; see Materials and Methods). In the first diallel (2010), plants were measured for dry panicle weight (DPW) and days to flowering; in 2011, the phenotyping was extended to include vegetative traits: leaf weight, stem weight, stem diameter and plant height (see Materials and Methods). The weighed panicles were further dissected to obtain levels of primary, secondary and tertiary branching (PBN, SBN and TBN, respectively), as well as rachis length, whorl number and primary branch length following Brown *et al*. [28]. Seed number was estimated based on DPW and seed dry weight (SDW) (see Materials and Methods), due to the high correlation (r = 0.88, *P*<0.001) found between DPW and total seed number within a subset of 60 different FLs and hybrids.

**Figure 3:**
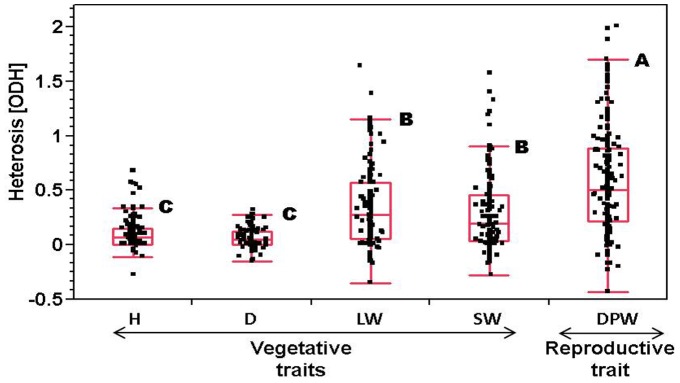
Overdominant heterosis (ODH) in the diallel. ODH distribution of vegetative (height, H; diameter, D; leaf weight, LW; stem weight, SW) and reproductive (dry panicle weight, DPW) traits. Different letters denote significant difference between ODH distributions (Kruskal-Wallis test, *P*<0.0001). Quantile boxes show the range between the 25th and 75th percentiles, including the 50th percentile indicated in between. The bottom and upper outer lines depict the 10th and 90th percentiles, respectively.

Different estimates of heterosis were computed for all traits: (1) mid parent heterosis, (2) BPH [35] and (3) ODH. The ODH parameter is a measure of the extent to which the phenotype of the progeny of a cross deviates from the phenotypic bounds of its parents, thus emphasizing overdominant or underdominant mode of inheritance (see Materials and Methods). Comparison of the traits’ ODH distribution showed significantly higher values for the reproductive trait DPW (median 0.51, quartile 0.216) than for any of the vegetative traits (Figure 3). Within the vegetative traits, leaf weight (median 0.27, quartile 0.05) and stem weight (median 0.19, quartile 0.035) showed significantly higher values than stem diameter (median 0.093, quartile 0) or plant height (median 0.068, quartile 0.01). Notably, a comparison of the reciprocal hybrids showed that there is one or no hybrid per trait which can be considered significantly different (*P*≤0.01). According to these results, the imprinting effect on heterosis was considered insignificant in our mapping populations.

Grain yield components such seed number and weight are known to be negatively correlated [36]. To determine whether these kinds of relations are also maintained at the heterosis level, the correlation pattern between the individual yield assemblers and DPW was compared to that between their heterotic modes of inheritance. At first, multiple regression analysis based on trait values found significant correlations between the three branching levels (PBN, SBN and TBN), SDW and total grain yield (DPW): the dominant effect was that of SDW (7.36, 0.04; effect and *P* value respectively), and a comparison of the effects of the different branching levels on DPW showed a stronger effect of SBN (4.78, <0.0001) relative to PBN (0.61, <0.001) and TBN (0.12, 0.014). Overall, this four-factor model explained 68% of the variation in grain yield (DPW). Next, a similar analysis between the heterosis values (BPH) of these traits showed that only SBN and PBN heterosis values are significantly and positively correlated with that of DPW (1.37, <0.0001; 0.396, 0.019, respectively), and together these two explained 35% of the DPW heterosis.

**Figure 4:**
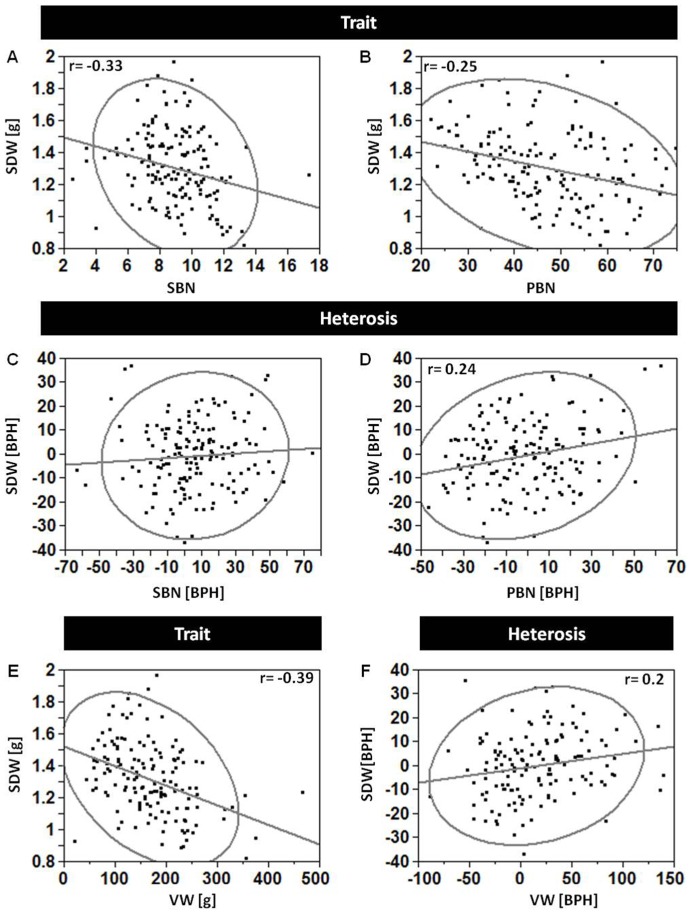
Hybrid reproductive superiority is induced by release of tradeoff relationship. Correlations between components of seed number (secondary branching number, SBN; primary branching number, PBN) and seed dry weight (SDW). Analyses of trait values show negative correlations (**A, B**) while analysis of heterotic values (best parent heterosis; BPH) show either no (**C**) or positive (**D**) correlation. **E–F.** Correlations between vegetative weight (VW) and seed dry weight (SDW). Analysis of trait values (**E**) shows negative correlation whereas that of heterosis values (**F**) shows positive correlation.

A similar comparison of the relationships within the grain yield components, both on the trait per se and the heterosis values, showed significant differences in the direction of the correlations between the two levels. Multiple regression analysis between the panicle architecture traits and seed number showed that SBN and PBN explain 65% of the variation in seed number across all hybrids in the 2011 experiment, with SBN showing an approx. eightfold stronger effect (275.5, <0.0001) than PBN (34.7, <0.0001). There was no significant correlation between TBN and seed number. A correlation test between trait values of SDW and these significant contributors for seed number indicated negative correlations for both SBN and PBN to SDW (r = −0.25 and −0.33; *P* = 0.0001 and 0.001, respectively; Figure 4A and 4B). On the other hand, analysis of the heterosis values showed no correlation between those of SBN and SDW (Figure 4C) and notably, a positive correlation between those of PBN and SDW (r = 0.24, *P* = 0.003, Figure 4D). A similar opposite pattern between traits’ relationships and their associated modes of inheritance was found with vegetative weight and SDW. While a significant negative correlation was found between the traits’ values (r = −0.39, *P*<0.0001, Figure 4E), their heterosis values were positively correlated (r = 0.2, *P* = 0.02; Figure 4F).

### Genomic Scan for HTLs

HTL mapping combines multiple alleles in the analysis and focuses on associations between specific allelic combinations and overdominant mode of inheritance. Therefore, the use of ODH rather than the classical mid parent heterosis or BPH [35] is not meant to describe all of the cryptic heterotic variation between two or more inbred parents but rather extract major loci which exhibit their overdominant effects over a wide genetic background. Optimal implementation of HTL mapping should include ultra-high-resolution genotyping of the inbred FLs, i.e. genotype by sequencing [37]. At this stage, we present the analysis using relatively low-resolution genotyping (a total of 85 genetic markers across 10 chromosomes; Table S2).

**Figure 5:**
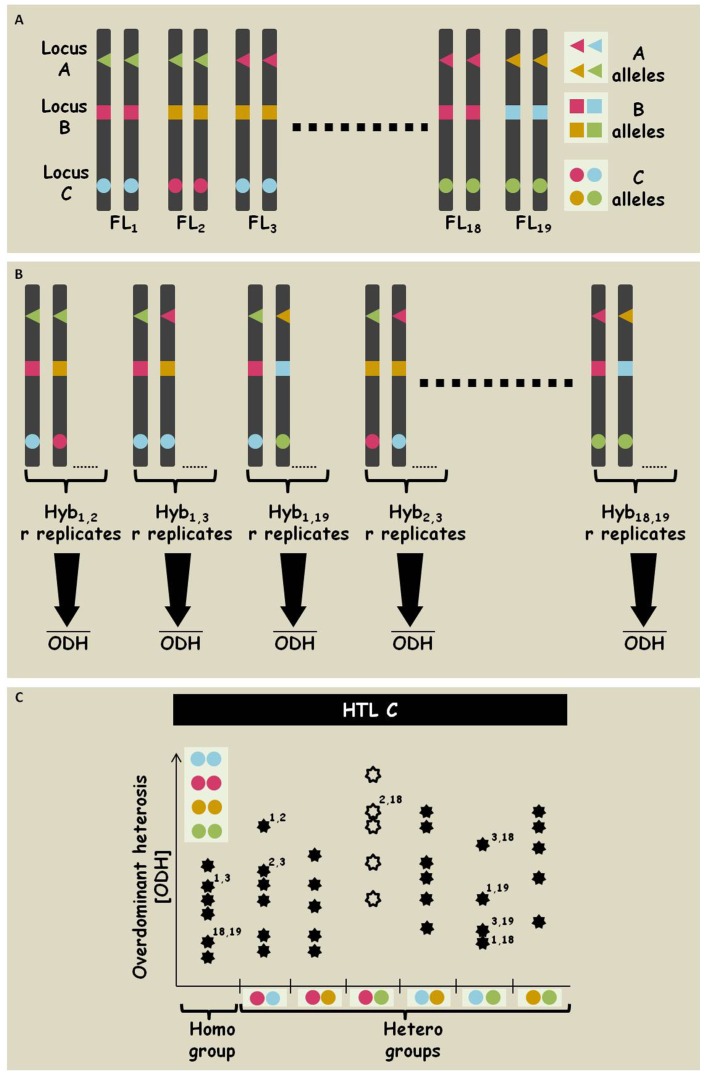
Illustration of the HTL mapping. **A.** Genotyping of the selected FLs is represented by three loci with different shapes (A, B and C), with each harboring 4 different alleles among the FLs of the diallel. **B.** Projection of the FLs genotype to the derived hybrids and calculation of the mean heterosis values (ODH) for the r replicates of an hybrid. **C.** Statistical analysis to identify specific hetero combinations with advantageous ODH values (the purple/green hetero-group in this illustration).

The nature of the mapping population and the homozygosity of the selected parents allowing to project the allelic state in each locus from the inbred parents onto their hybrids (Figure 5A and 5B). In each locus, the hybrids are sorted to the different genotypic groups: the different heterozygous combinations are treated as separate hetero-genotypic groups, and the homo-genotypic group includes hybrids that are homozygous for the different alleles (In this study, due to the relatively high number of rare alleles in each locus, which is characteristic of SSR markers, the homozygotes in each locus were grouped together; Figure 5C). Next, the mean ODH values of each hybrid were considered (Figure 5B) and the distributions of these values were compared between genotypes to associate specific allelic interactions with overdominance (see Materials and Methods). A locus was considered a HTL if at least one heterozygous combination showed significantly advantageous heterosis values as compared to the homozygous combinations in both years (Figure 5C). Overall, this analysis revealed three significant HTLs for grain yield (DPW) residing on chromosomes 1 and 4 (namely *hDPW1.1, 1.2,* and *4.1*; Figure S3). Overall, these loci represent 3.5% of the loci tested.

A structured population is a known limitation when performing association mapping [5]. We therefore examined whether the clustering pattern of the FLs bias the mapping results due to co-variation of heterosis levels and FL assignment to genomic groups. Sorting the FLs into four groups ([31], K = 4, Figure 2) allowed determining the genomic combinations for each hybrid. When this new variable was considered a co-factor in each of the two steps of the mapping procedure (see Materials and Methods), the results remained the same. All three, and only those three previously identified HTLs, were consistently significant over 2 years.

### Analysis of HTLs for Grain Yield in Consecutive F2 Generation

**Figure 6:**
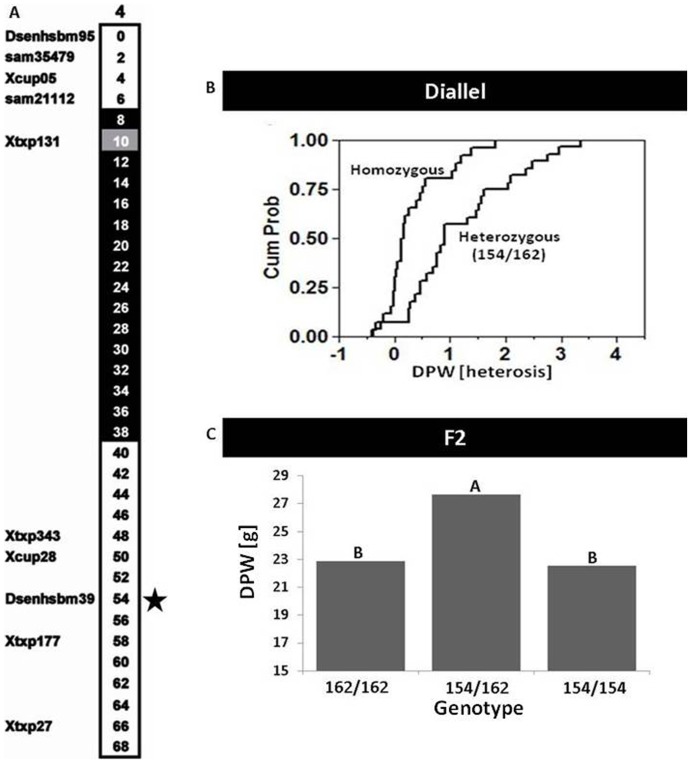
The *hDPW4.1* grain yield heterotic trait locus (HTL). **A.** Chromosomal location of the Dsenhabm39 SSR marker is indicated by star on the physical map of chromosome 4. Black and white coloring indicate pericentric-heterochromatic and telomeric-euchromatic chromosomal regions, respectively. Gray indicates markers within pericentric-heterochromatic chromosomal regions. **B.** Cumulative distribution function plot showing the ODH values of the significant hetero-genotypic (154/164) and homo-genotypic (H:H) groups for the same marker in the diallel (year 2011). **C.** Linkage analysis of the *hDPW4.1* locus with dry panicle weight (DPW) in the F2 population. Different letters above bars denote significant difference (*P*<0.05; Hsu’s MCB test) between mean values.

Perhaps the major concern with the HTL mapping approach is possible confounding effects, i.e. the association of certain local intra-locus interactions to the heterotic mode of inheritance where in fact this association is due to either a linked or non-linked locus found in linkage disequilibrium. This scenario is best illustrated by the example of loci in which the FLs are sorted in an identical segregation. This will lead to false associations between certain heterogeneities and the heterotic mode of inheritance where in fact, only one of these loci carries the functional causal polymorphism. We therefore tested the validity of the causal relationship identified between one of the mapped HTLs and the overdominant mode of inheritance in consecutive generations. One of the hybrids participating in the 2010 diallel (cross between SB018 and SB153, Figure 2) was caged to obtain F2 progeny. These plants were planted in the 2011 trial for genetic analysis of *hDPW4.1* (Figure 6A), which showed a significant association between heterozygosity for the 154/162 allelic combination and increased ODH values for DPW in the diallel in both years (Figure 6B). All F2 progeny were genotyped with the Dsenhabm39 marker and analyzed for their DPW. A comparison of the grain yield values of the three genotypic groups showed that the mean DPW of plants carrying the two specified alleles of *hDPW4.1* (154/162) was significantly higher than those of both homozygous plants (154/154 and 162/162, respectively; *P* = 0.048 and *P* = 0.037; Hsu’s multiple comparisons with the best test), with a mean effect of 21% (Figure 6C). These results thus support the overdominant mode of inheritance associated with this allelic combination, as suggested by the original HTL mapping (Figures 6A, and S3).

### Contribution of HTLs to Heterotic Variation and Their Epistatic Interactions

**Figure 7:**
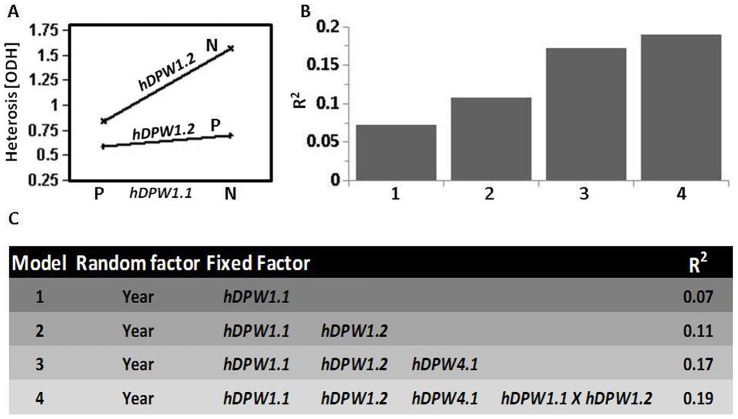
Contribution of heterotic trait loci and their epistatic interactions to heterotic variation in a diallel population. **A.** Heterosis least squared means plot. The x axis represents the genotypes of *hDPW1.1*. The lines represent different genotypes of *hDPW1.2.* N, neutral non-interacting genotypic allelic combinations; P, positive interacting combination. **B.** Accumulated variation explained by the model (*R^2^)* with each additional factor. **C.** The factors included in each of the models.

We next investigated the contribution of each HTL to the overall heterotic variation found in the diallel population, as well as the possible epistatic interactions between the three loci. Due to the multiple-parent population structure and the nature of the SSR markers, a direct two-way analysis is in fact unattainable. For this reason, in each of the identified HTLs the interacting allelic combinations were considered to be one genetic group (P; positive), and all other combinations were treated as a single neutral non-interacting combination (N). *hDPW4.1* showed the highest contribution to heterosis (12%; *P*<0.0001) as a single factor, and *hDPW1.1* and *hDPW1.2* contributed 7% and 4% (*P*<0.0001 and *P*<0.0026), respectively, to this mode of inheritance. Next, two-way interactions were analyzed to test for epistatic relationships between the different loci. Of the three possible interactions, only that between the two HTLs on chromosome 1 was significant (*P* = 0.014; Figure 7). In addition, to estimate the cumulative contribution of all HTLs to the overall heterotic variation, a three-way linear regression was performed (Figure 7B and 7C). Overall, this final model explains 19.0% of the heterotic variation found in this population (Figure 7B and 7C).

## Discussion

### HTL Mapping and its Implications for Rapid Identification of Overdominant Loci

The search for the genetic architecture of quantitative and complex phenotypes in plants has experienced a quantum leap in the depth of genomic architecture descriptions [38], as well as in the speed of unraveling causative polymorphisms. This is mainly due to the adoption of population-based association approaches [39], [40] and application of higher throughput resequencing and marker genotyping [41]. In this study, we address the challenge of developing new genetic mapping strategies to direct and optimize the discovery of genetic variation [1] underlying heterosis, which is a most important component in modern breeding.

Although the diallel population does not offer the advantage of permanent populations, such as the ability to propagate it and phenotype each genotype under different environments, there are several attributes which make it an attractive alternative for unbiased discovery of novel allelic variation in plant genetic resources. It is important to note that the identification of hitherto unknown heterosis loci in this study was achieved with no prior knowledge of the level of heterosis or its genetic basis. While this may limit the validation of our results, no comparable heterosis map for sorghum exists; however, we do show that for *hDPW4.1*, the overdominant mode of inheritance is maintained in a consecutive F2 population (Figure 6). Although mapping heterosis loci might be simpler using diallelic populations such as F2 crosses, advanced inter-crosses and recombinant inbred lines descended from two parents, such an approach requires a priori knowledge of the mode of inheritance of their hybrid for the different traits analyzed. The multi-parent and multi-allelic system characteristic of the mapping population, in fact, increases the likelihood of detecting allelic combinations with synergistic effects on yield, or on any other trait. Nevertheless, this relatively rapid mapping procedure, including the harvesting of allelic variation into a single population of hybrids, raises both operative and statistical challenges. Given that the power to compare the heterotic values of any allelic combination at each locus relies on a minimal genotypic frequency in the hybrids (Figure S4), the choice of FLs from a wide collection should attempt to provide a balanced representation for the different alleles. Such a representation could be achieved by simulating the different FL combinations to achieve an optimal allelic combination in most of the loci tested. This might be achieved, as in this study, by relatively low-resolution genotyping. In practice, such selection should be well thought out to include the possibility of obtaining all hybrids required for the HTL mapping in a reasonable time. Therefore, in this study, relevant characteristics for sorghum such as day-length sensitivity were also considered (data not shown).

The basic premise underlying the HTL mapping approach is that intra-locus interactions play a significant role in the manifestation of heterosis. The genetic analysis of each locus within a diallel population is highly subjected to epistatic interactions due to the genome-wide intersection of many genetic backgrounds and, although each locus is analyzed separately (single-point analysis), the HTL mapping results reflect the influence of inter-allelic interactions as well. Despite the fact that this genetic “background noise” may increase intra-genotypic-group variation (Figure S2), thereby reducing the power of discrimination between the phenotypic distributions of the different groups, we hypothesize that the major loci will still emerge. Moreover, in our opinion, such noise is biologically relevant due to the significant role of these interactions in manifesting complex phenotypes [26], [42].

This study differs from the analysis of heterosis in a diallel population reported previously by Cho *et al*. [15] in that (1) the different hetero-genotypic groups were separated and the allelic combination mediating the heterosis in each locus was specified, (2) the heterosis was analyzed with values that emphasize overdominant mode of inheritance (ODH), and (3) a less permissive permutation approach was used to set the genome-wide type I error. This probably explains the smaller proportion of markers associated with ODH in this study (Figure S3), as compared to over 30% positive associations in Cho *et al*.’s [15] analysis. Notably, the analysis of sorghum heterosis in this study, although separating the hetero-genotypic groups, treats the homozygous hybrids in each locus as a single reference group. This originates from the hypothesis that homo-allelic combinations will not result in synergistic or antagonistic interactions. It is also possible to further separate the homo-genotypic group and compare each hetero-genotype to hybrids carrying each of the two interacting alleles in a homozygous state (such an approach is currently being tested in another study with *Saccharomyces cerevisiae* hybrids; Ben-Israel and Fridman, unpublished results).

It should be noted that as a methodology, the HTL mapping approach can also be used to map the opposite trend in hybrids, i.e. to scan the genome for intra-locus interactions associated with hybrid inferiority. Whereas in our study, the general positive ODH values for all of the traits (Figure 3) directed HTL mapping for overdominant loci, recent studies have shown that such interactions at a single gene play key roles in the evolution of plant populations and mediate an underdominant mode of inheritance [43]. It would therefore be beneficial to implement the HTL mapping approach in studies aimed at identifying ‘genes of speciation’ in models such as *Arabidopsis thaliana*, in order to test the prevalence of additional loci governing speciation.

### Significance of Intra-locus Interactions in Heterosis

Despite the fact that the HTL mapping was performed with a relatively small number of genetic markers, it allowed us to apply a novel mapping approach to study the relative contribution of intra-locus interactions to the heterotic variation in the gene pool. Overall, at this resolution, we identified three HTLs which were reproducibly associated with grain yield heterosis over 2 years, and additional loci which were not considered because the effect was only found in one growing season (data not shown). A comparison of our results to other studies shows that the number of loci with reproducible and significant overdominant effects on grain yield is comparable to that found in rice, for example. Although QTL studies show that heterosis is controlled by a large number of loci, and includes the involvement of complex epistasis, the number of true overdominant loci for grain yield with reproducible effects in more than 1 year sum to less than 4% of the markers used [44], [45]. Based on the analysis in our study, it can be concluded that the portion of heterosis explained by the overdominant model [7] is significant, contributing approximately 20% to the overall heterotic variation. This value is either an underestimate of the effects of intra-locus interactions due to the low resolution of the mapping, or it might suggest that the dominance genetic model is highly prevalent. In addition, the ability to identify significant HTLs in this study implies that, as in standard models for quantitative variation, there may be a small number of major loci underlying heterosis (the overdominant ones), and a large number of loci which make a small contribution following the infinitesimal genetic model [46].

### *hDPW4.1*: HTL for Grain Yield in *S. Bicolor*

This study provides the identification of grain yield heterotic loci in a major crop plant, through genetic association over a wide genetic background, all in a relatively short time. Starting from a wide collection (Figure 1 and Table S1), the HTL mapping approach pinpointed three significant loci associated with heterosis. Furthermore, the superiority of heterozygosity for *hDPW4.1* in an F2 population (Figure 6), despite possible masking effects of inbreeding and epistatic interactions from the rest of the genome, strongly support this association obtained in the diallel. Further phenotypic and genetic analyses are required to determine the nature of this locus, which increases grain yield by more than 20%. This will include looking for pleiotropic effects which would support a multiplicative model [47], and perhaps the more challenging task of identifying and defining the causal polymorphisms underlying these effects. This will require fine mapping of this locus, either by screening for recombination events in large F2 populations, followed by phenotypes of derived F3 progeny, or alternatively, selecting for relevant recombined haplotypes in recombinant inbred populations. In sorghum, as in maize, these populations have become a key component in dissecting additive variation using the nested-association mapping (NAM) approach [48], [49]. Therefore, once the ultrahigh genotype of these FLs will be determined using same approaches it will be imperative to compare the haplotype structure between these to the sorghum NAM founder lines. This may assist in zooming in on these HTLs at high resolution and determining the genetic factors that mediate these major heterotic effects.

### *S. Bicolor*: an old-new Model for Studying Heterosis

There are several genetic and developmental attributes of *S. bicolor* which position this crop plant as an ideal model system for in-depth dissection of heterosis, and for projecting the outcome of these studies on its relatives in grasses. Genetic analysis of the wide core collection of *S. bicolor* showed two main genetic attributes. First, the amount of allelic richness found in this material far exceeds that found in other cultivated crops; it is, in fact, comparable to that of the wild ancestors of other major crops. For example, recent analysis of a large core collection of both cultivated and wild barley accessions (*Hordeum vulgare* and *H. spontaneum*, respectively–The Barley1K) with a similar genetic marker system showed the significant genetic bottleneck experienced by this crop during domestication, including reduction in allelic richness from 13.4 to 6 alleles per locus [23], [50]. In the cultivated *S. bicolor*, on the other hand, the allelic richness–which is a prerequisite for testing multiple allelic interactions in a population structure such as the one presented in this study–is in fact comparable to that of *H. spontaneum*: more than 13 alleles per locus in the wide collection and 7 alleles per locus in the FLs of the diallel. In addition, while there is ample genetic heterogeneity in the wide collection, each of the accessions is, by itself, highly homozygous, reflecting the partial allogamous mode of reproduction in this plant [17]. These two attributes are critical in the implementation of HTL mapping since they provide the opportunity to extract a rich allelic and phenotypic repertoire (Figure 1) while retaining the ability to project the genotype of the hybrids from their fully homozygous parents (Figure 5).

Notably, comparison of the sorghum DPW HTL mapping results with previous QTL studies for grain yield in maize [9], [12], [20] shows synteny of two sorghum HTLs with maize bins that were reported to harbor overdominant QTLs (Figure S5). On average, the syntenic blocks on sorghum corresponded to a threefold larger block in maize and in addition, *hDPW4.1* was syntenic with two heterotic maize QTLs for yield that reside on bins 2.04 and 10.04 (Figure S5). These observations reflect the evolutionary history of maize and sorghum with approximately 3× genome expansion in maize since its divergence from sorghum 12 MYA. They lend further support to the usefulness of the latter for associating causative polymorphism with complex traits due to reduced genomic and possibly functional redundancy between paralogs.

### Possible Mechanisms Governing Grain Yield Heterosis in Sorghum

The wide diallel used in this study creates a unique phenotypic and genetic framework for a systematic analysis of the transition from inbreds to hybrids, and of how hybrid superiority over inbreds is orchestrated via the different developmental pathways in the plant. Smith and Fretwell [51] predicted that once the resources available for reproduction are fixed, a tradeoff between seed size and number is inevitable: any increase in the size of individual offspring must be compensated for by a reduction in offspring number. Indeed, negative correlations were found in this study between grain yield components across the hybrid populations and these results are in agreement with previous studies [37]. This was observed for reproductive assemblers–seed number and seed weight (Figure 4A and 4B), and for productive competitors–vegetative weight and seed weight accumulation (Figure 4E). To answer the question of whether hybrids can surpass their inbred parents for two traits under such a tradeoff relationship, correlation analyses using the heterosis values of both traits were conducted. There was a lack of correlation between heterosis values of SBN and SDW (Figure 4C), as compared to positive correlations between those of PBN and SDW (Figure 4D) and between vegetative and reproductive heterosis (Figure 4F). These differences between trait values and their associated heterotic mode of inheritance suggest that introduction of new allelic combinations within hybrids eliminated the limiting intra-plant compensatory tradeoff. The new genomic pattern in the hybrids can be interpreted as an environment with better growth potential for the plant compared with their inbred parents [52]. Developmental and biochemical reasons for the hybrid’s ability to increase both vegetative and reproductive output compare to its inbred parents indicate that either the efficiency of the whole system is much higher, or rate-limiting factors are modulated due to hybridization.

More specifically, the mechanism underlying such relaxation of metabolic flow or increase in growth potential may originate from changes in the activities of gene products with critical spatial and temporal distribution, at the cellular level, at early developmental stages or both. Such a sequential developmental pathway underlying naturally occurring variation, though not modulated by inter-allelic interactions, was exemplified in the previously characterized tomato QTLs *fw2.2* and *Brix9-2-5*. In those studies, it was found that changes in a single gene (*ORFx* or the apoplastic invertase *LIN5*) occurring early in ovary development lead to increases in cell number or total sugar yield in the mature fruit [53], [54]. It would therefore be interesting to determine the causative polymorphism underlying the overdominant effects of the HTLs and (1) test whether these genes share similar early effects on plant development that lead sequentially to increased growth and if so, (2) whether overdominant QTLs have unique characteristics relative to additive QTLs (see above), and eventually (3) determine the molecular and biochemical bases for the differences between homo-alleles and hetero-alleles, the stoichiometry of their products, and how this relates to modulation of the reproductive output.

## Supporting Information

Figure S1 **Scheme of the two mapping populations from A. 2010 and B. 2011 experiments.** Dark and light purple blocks show crosses for which accessions indicated on the left or bottom, respectively, were used as females. Blue blocks show hybrids for which two reciprocal crosses were analyzed. Brown indicates hybrids whose two reciprocal crosses were field-trialed, but there were not enough replicates (n <3 for each) for statistical comparison between them. White blocks indicate missing hybrids.
(TIF)

Figure S2 **Heterotic trait locus (HTL) mapping.** Two-step genomic scan as performed with overdominant heterosis (ODH) values derived from the 2011 field experiment, shown for representative markers. **A.** Dsenhsbm99 showing similar ODH distributions for the different genotypic groups, i.e. did not pass the first mapping step (GLM). The line across each diamond and the vertical span represent the group mean and the 95% confidence interval for each group, respectively. **B.** Xcup64 showing significant difference (GLM, perm. *P* = 0.002) between ODH values of the different genotypic groups, albeit with no advantage for specific hetero-genotypic group as compared to the homo-genotypic group (H:H). This marker passed the first step and failed in the second (Kolmogorov-Smirnov). **C.** Dsenhsbm40 that passed the first step (GLM, perm. *P* = 0.005) and the second step with a significant advantage only for the hetero-genotypic groups 154∶156 and 154∶164 (in gray) vs. the homo-genotypic group (H:H; Kolmogorov-Smirnov test, *P* = 0.0008, 0.0165, respectively). **D.** Cumulative distribution function plot showing the ODH values of the significant hetero-genotypic (154∶156, 154∶164) and homo-genotypic groups (H:H) for the same marker (Dsenhsbm40). DPW, dry panicle weight.
(TIF)

Figure S3 **Chromosomal physical map (in Mbp) of markers used in this study and the identified heterotic trait loci (HTLs) for grain yield (dry panicle weight; DPW).** Position is only shown for HTLs that were mapped over 2 years–each HTL is marked with a black diamond. Black and white coloring indicate pericentric-heterochromatic and telomeric-euchromatic chromosomal regions, respectively. Gray indicates markers within pericentric-heterochromatic chromosomal regions.
(TIF)

Figure S4 **Illustration of the allelic distribution in a diallel.** **A.** Allelic distribution in founder lines (FLs) including the common A1 and A2 alleles shared by 4 and 3 parents, respectively, and the rare A3 allele found in FL18 and FL19. A rare allele is defined as one carried by less than three FLs. The genotype of each hybrid is projected from the two homozygous parents. **B.** Six homozygous hybrids derived from the four FLs that share the A1 allele (homo-genotypic group A1/A1). **C.** Eight heterozygous hybrids derived from four FLs that share the A1 allele and two FLs that share the A3 allele (hetero-genotypic group A1/A3). **D.** A rare homo-genotypic group (A3/A3) including a single hybrid derived from a cross between FLs sharing the rare A3 allele. This group cannot be compared to the corresponded hetero-genotypic groups (any groups that harbor one copy of A3).
(EPS)

Figure S5 **Comparative heterosis mapping of sorghum and maize.** For each sorghum heterotic trait locus (HTL; white diamonds), genomic intervals which are syntenic with the maize genome are shown. The locations of the maize overdominant QTL for yield-associated traits (kernel number, KN; grain yield, GY) on the maize bin map are drawn based on a combination of data from Tang *et al*. [9], Cockerham and Zeng) [12] and Frascaroli *et al*. [20].
(EPS)

Table S1 **List of the accessions included in the wide Sorghum bicolor sps. bicolor collectionTable S2: SSR markers used in this study.**
(XLSX)

Table S2 **SSR markers used in this study.**
(XLSX)
