# Supplementary figures and images for: Heterotic Trait Locus (HTL) Mapping Identifies Intra-Locus Interactions That Underlie Reproductive Hybrid Vigor in Sorghum bicolor
Source: PLoS One. 2012 Jun 25;7(6):e38993. doi: 10.1371/journal.pone.0038993 (PMC3382592; doi:10.1371/journal.pone.0038993)

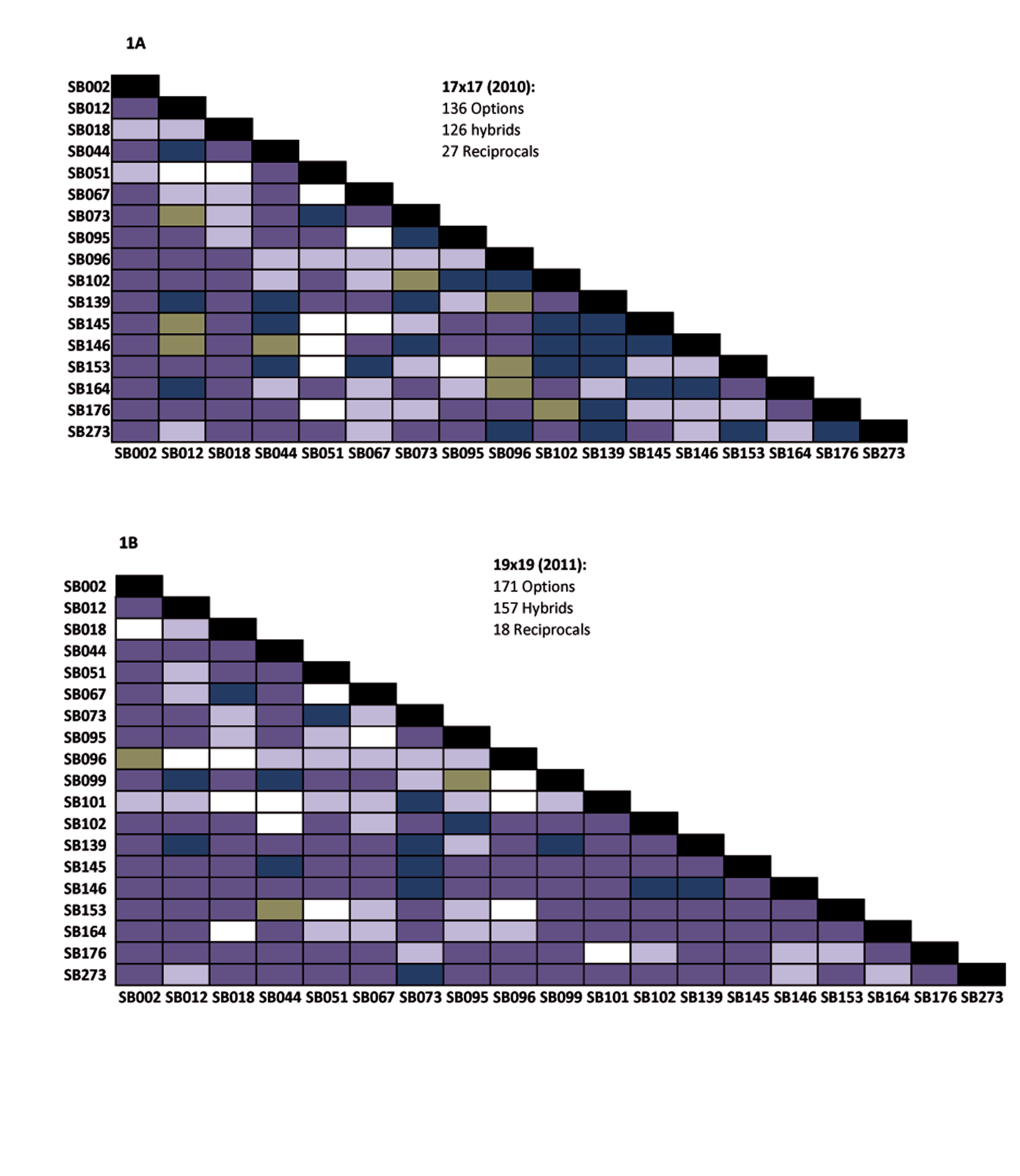

Supplement: Figure S1 — Scheme of the two mapping populations from A. 2010 and B. 2011 experiments. Dark and light purple blocks show crosses for which accessions indicated on the left or bottom, respectively, were used as females. Blue blocks show hybrids for which two reciprocal crosses were analyzed. Brown indicates hybrids whose two reciprocal crosses were field-trialed, but there were not enough replicates (n <3 for each) for statistical comparison between them. White blocks indicate missing hybrids. (TIF) [file pone.0038993.s001.tif]

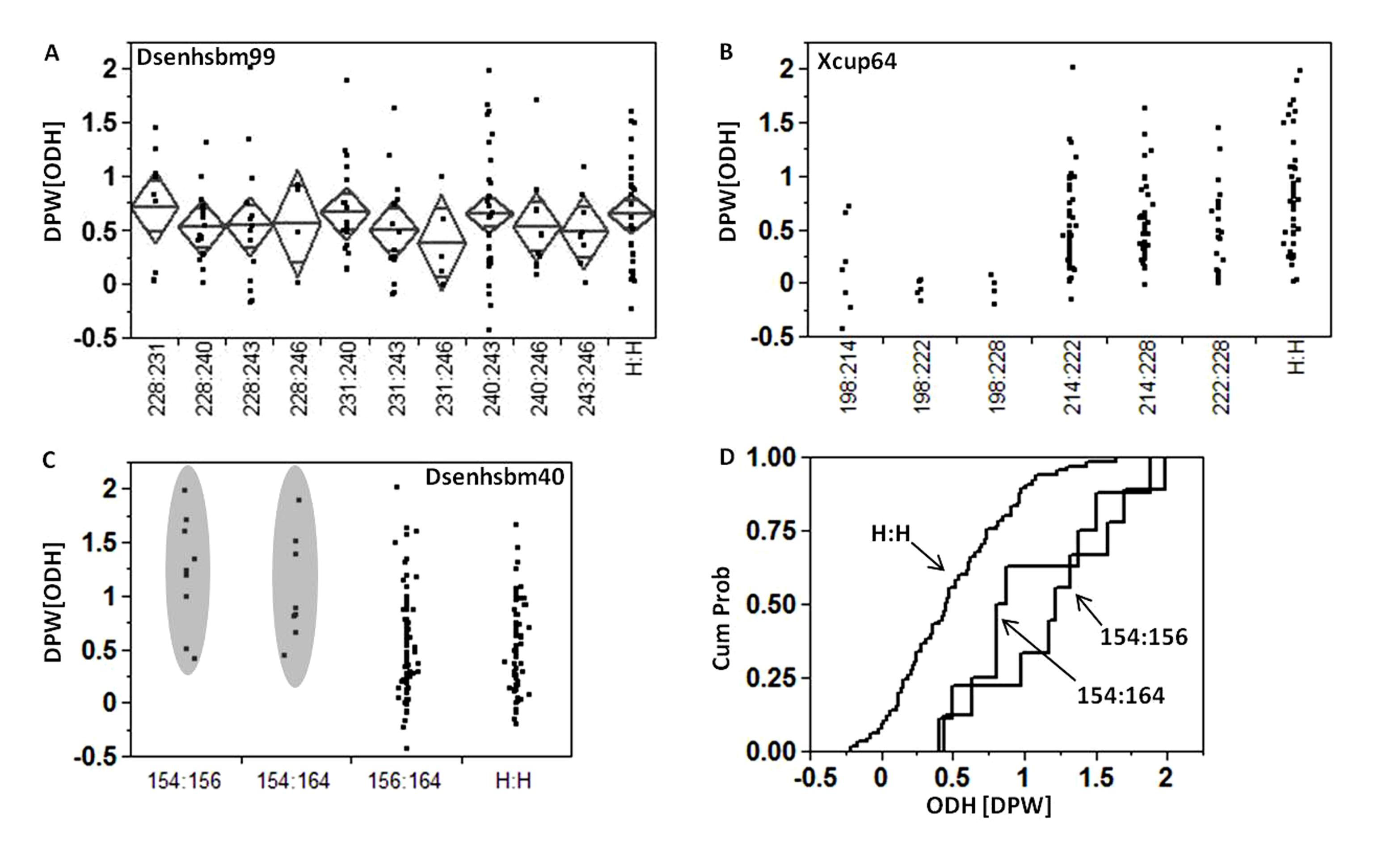

Supplement: Figure S2 — Heterotic trait locus (HTL) mapping. Two-step genomic scan as performed with overdominant heterosis (ODH) values derived from the 2011 field experiment, shown for representative markers. A. Dsenhsbm99 showing similar ODH distributions for the different genotypic groups, i.e. did not pass the first mapping step (GLM). The line across each diamond and the vertical span represent the group mean and the 95% confidence interval for each group, respectively. B. Xcup64 showing significant difference (GLM, perm. P = 0.002) between ODH values of the different genotypic groups, albeit with no advantage for specific hetero-genotypic group as compared to the homo-genotypic group (H:H). This marker passed the first step and failed in the second (Kolmogorov-Smirnov). C. Dsenhsbm40 that passed the first step (GLM, perm. P = 0.005) and the second step with a significant advantage only for the hetero-genotypic groups 154∶156 and 154∶164 (in gray) vs. the homo-genotypic group (H:H; Kolmogorov-Smirnov test, P = 0.0008, 0.0165, respectively). D. Cumulative distribution function plot showing the ODH values of the significant hetero-genotypic (154∶156, 154∶164) and homo-genotypic groups (H:H) for the same marker (Dsenhsbm40). DPW, dry panicle weight. (TIF) [file pone.0038993.s002.tif]

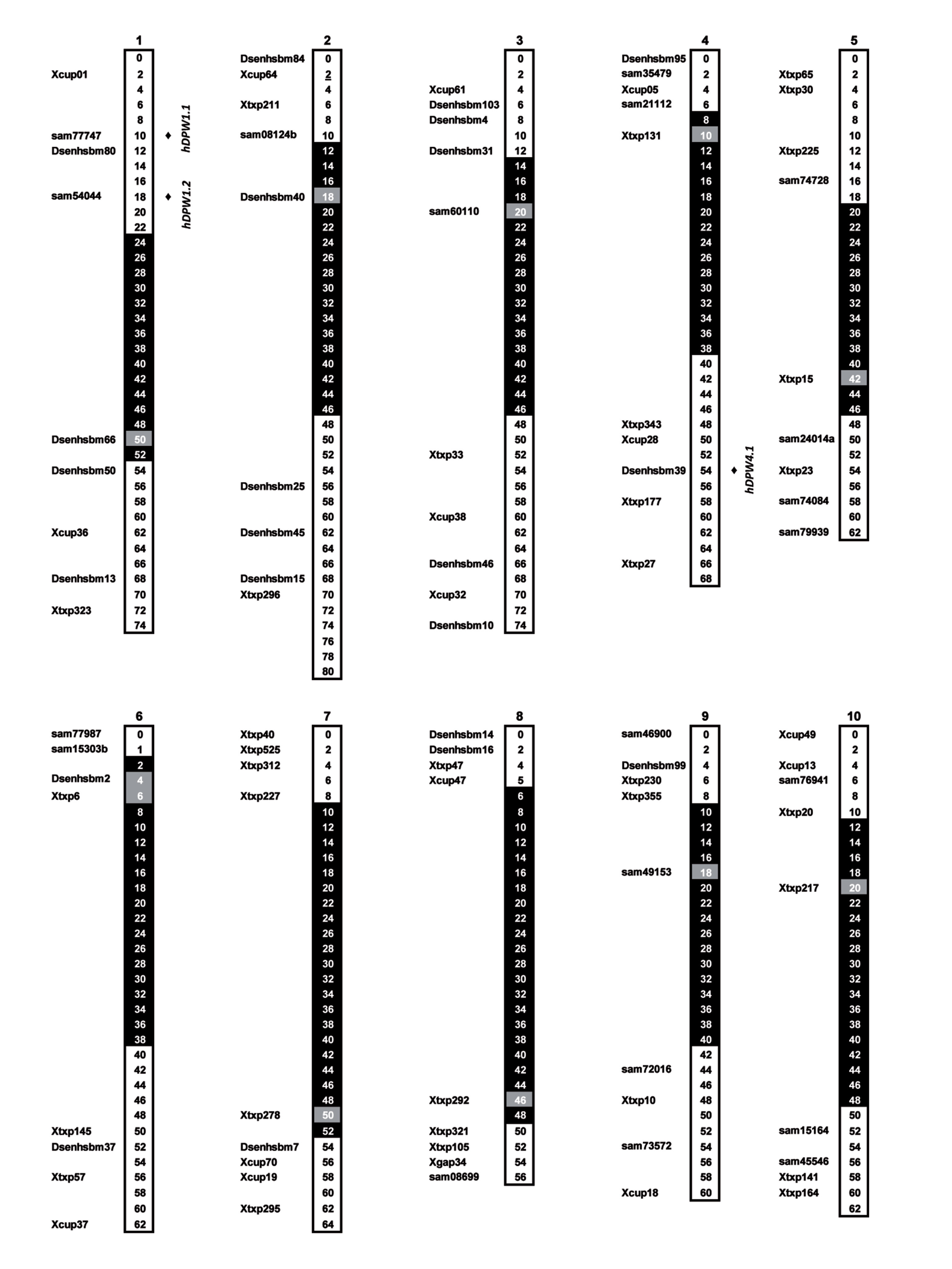

Supplement: Figure S3 — Chromosomal physical map (in Mbp) of markers used in this study and the identified heterotic trait loci (HTLs) for grain yield (dry panicle weight; DPW). Position is only shown for HTLs that were mapped over 2 years–each HTL is marked with a black diamond. Black and white coloring indicate pericentric-heterochromatic and telomeric-euchromatic chromosomal regions, respectively. Gray indicates markers within pericentric-heterochromatic chromosomal regions. (TIF) [file pone.0038993.s003.tif]
